# Plasma Levels of Brain-Derived Neurotrophic Factor and S100B in Relation to Antidepressant Response to Ketamine

**DOI:** 10.3389/fnins.2021.698633

**Published:** 2021-07-20

**Authors:** Haitang Jiang, Emma R. Veldman, Mikael Tiger, Carl-Johan Ekman, Johan Lundberg, Per Svenningsson

**Affiliations:** ^1^Department of Psychosomatics and Psychiatry, Zhongda Hospital, Medical School of Southeast University, Nanjing, China; ^2^Department of Clinical Neuroscience, Karolinska Institutet, Stockholm, Sweden; ^3^Centre for Psychiatry Research, Department of Clinical Neuroscience, Karolinska Institutet and Stockholm Health Care Services, Stockholm, Sweden

**Keywords:** ketamine, mBDNF, S100B, major depressive disorder, neurotrophin

## Abstract

**Background:**

Evidence demonstrates that brain-derived neurotrophic factor (BDNF) and S100 calcium-binding protein B (S100B) have a pivotal role in the pathogenesis of major depressive disorder (MDD) and they are proposed as predictors of antidepressant response. Ketamine produces rapid antidepressant effects in MDD and pre-clinical studies suggest the necessity of increased BDNF levels for the antidepressant action of ketamine. However, studies observing the change of blood BDNF levels after ketamine intervention are inconsistent and studies about the role of plasma S100B in ketamine administration in MDD patients are lacking.

**Method:**

We evaluated mature BDNF (mBDNF), S100B levels in plasma and their associations with depression severity in 30 Selective Serotonin Reuptake Inhibitor (SSRI)-resistant MDD patients enrolled in a randomized controlled trial of ketamine compared (*n* = 20) to a placebo (*n* = 10) control (saline). Severity of depression was assessed using the Montgomery–Åsberg Depression Rating Scale (MADRS).

**Results:**

Plasma mBDNF and S100B were not significantly changed after 1–2 days of single ketamine compared to placebo. Plasma mBDNF and S100B levels did not significantly differ in responders compared to non-responders of ketamine treatment. The change of plasma mBDNF levels was positively correlated with the improvement of MADRS score after 1–2 weeks of open-label ketamine treatment (rho = 0.495, *p* = 0.031), though this change did not survive correction for multiple comparisons.

**Conclusion:**

These findings do not support the hypothesis that ketamine treatment increases BDNF plasma levels in MDD patients. No effect of ketamine treatment on S100B plasma levels was seen.

## Introduction

Major depressive disorder (MDD) is the most common psychiatric disorder, affecting more than 300 million people, and the major cause of disability and suicidal death globally (World Health Organization, 2017). Conventional antidepressant treatment based on the monoamine hypothesis needs weeks to months before the onset of a clinical effect. One-third of MDD patients fail to respond to multiple antidepressant treatments, a fate so common it has been given a label of its own: treatment-resistant depression (TRD) (Trivedi et al., 2006). Thus, there is a major unmet medical need for more effective and rapid-acting therapies. Ketamine, an N-methyl-D-aspartate receptor antagonist, was demonstrated to have rapid antidepressant efficacy (Berman et al., 2000), including in MDD patients who are otherwise treatment resistant.

Neurogenesis is thought to play an important role in the anti-depressant action of ketamine (Duman et al., 2012), therefore neurotrophic factors, such as brain-derived neurotrophic factor (BDNF), may be involved in ketamine’s mechanism of action. BDNF is a neurotrophin that is important in the regulation of neurite outgrowth, functional neuronal connections, synapse formation, and mediating synaptic plasticity (Castrén et al., 2007; Autry and Monteggia, 2012; Lindholm and Castrén, 2014; Castrén and Kojima, 2017). BDNF arises from a precursor protein, proBDNF, which may be further cleaved to mature BDNF (mBDNF) within the Golgi or vesicles by furin or pro-protein convertases, or in the synaptic cleft by extracellular proteases (Lu et al., 2005; Hashimoto, 2007, 2010; Dieni et al., 2012). mBDNF binds with high affinity to the tyrosine kinase receptor, exerts neurotrophic activity, and anti-depressive effects, whereas proBDNF binds preferentially to p75NTR and has opposite functions. Numerous studies implicate a role of BDNF in MDD and antidepressant drug action in general (Polyakova et al., 2015; Yang et al., 2020; Duman et al., 2021). BDNF is also implicated as a critical component of the antidepressant action of ketamine (Autry and Monteggia, 2012; Duman et al., 2012). Ketamine rapidly increases BDNF expression in hippocampus (Autry et al., 2011; Ma et al., 2017) and the BDNF-TrkB-dependent differentiation of hippocampal neurons (Ma et al., 2017). Ketamine fails to exert antidepressant actions in conditional BDNF knockout (Autry et al., 2011) and mice with the BDNF Val66Met polymorphism (Liu et al., 2012), which impairs the activity-dependent secretion of BDNF (Egan et al., 2003). Intra-mPFC infusion of an anti-BDNF neutralizing antibody blocks ketamine’s antidepressant effects (Lepack et al., 2014). In addition, MDD patients with a BDNF Val66Met allele are less likely to respond to ketamine (Laje et al., 2012). Collectively, these findings suggest that increased BDNF levels are required for the antidepressant actions of ketamine. However, studies investigating blood BDNF levels in MDD patients receiving ketamine treatment show inconsistent results. While increased plasma BDNF levels after ketamine intervention in MDD patients have been repeatedly reported (Duncan et al., 2013; Haile et al., 2014), other studies point toward a lack of effect (Machado-Vieira et al., 2009; Medeiros et al., 2021). Therefore the role of BDNF in the anti-depressant actions of ketamine remains elusive and additional well-controlled trials are of interest.

S100 calcium-binding protein B (S100B) is found primarily in astrocytes. The protein can easily cross the blood-brain barrier (BBB) and ends up rapidly in the systemic circulation. S100B protein has thus been suggested as a candidate peripheral biomarker of astrocyte activation and/or permeability of the BBB and injury of the central nervous system (Gonçalves et al., 2008). At nanomolar concentrations, S100B promotes cell survival, proliferation and differentiation, while micromolar concentrations, which occur during exposure to stress, triggers inflammation by binding to the receptor for advanced glycation end products (RAGE) (Donato et al., 2013). There is evidence for dysregulated S100B levels in MDD and a relation to antidepressant treatment: in an animal model of depression, brain S100B levels were shown to be increased, which could be decreased by conventional antidepressants (Wang et al., 2016). Our previous studies showed that overexpression of S100B caused depressive-like behaviors in mice (Kindlundh-Högberg et al., 2009), which may involve hyperactivation of serotonin 5-HT7 receptors (Stroth and Svenningsson, 2015). Moreover, S100B has been suggested to serve as a biomarker that predicts behavioral responses to chronic fluoxetine treatment (Benton et al., 2012). In humans, peripheral levels of S100B have shown to be elevated in MDD patients (Arolt et al., 2003; Schroeter et al., 2013; Shi et al., 2020) and associated with depression severity (Uher and Bob, 2012; Schroeter et al., 2013). Moreover, clinical reports show a decrease in plasma S100B levels after antidepressant treatment (Arolt et al., 2003; Schroeter et al., 2013) and a predictive role of S100B levels for response to treatment with venlafaxine, imipramine, and other antidepressant treatments (Arolt et al., 2003; Ambrée et al., 2015; Shi et al., 2020). However, no study exists about the regulation or role of S100B upon ketamine administration in MDD patients.

In our study, we examined the relationship between plasma levels of mBDNF and S100B and depression severity, obtained during a randomized, placebo-controlled trial of ketamine in Selective Serotonin Reuptake Inhibitor (SSRI)-resistant MDD. The aim of this study was (i) to determine if mBDNF and S100B plasma levels were changed after ketamine treatment and (ii) to evaluate the relationship between clinical outcomes and levels of these plasma proteins in SSRI-resistant MDD patients.

## Materials and Methods

The present study was conducted as part of a randomized placebo-controlled clinical trial of ketamine compared to saline in patients with SSRI-resistant MDD [see European Union Drug Regulating Authorities Clinical Trials Database (EudraCT: 2017-003405-17)]. The primary study, which investigated serotonin receptor densities using Positron Emission Tomography (PET), was previously published (Tiger et al., 2020). The study was approved by the Regional Ethical Review Board in Stockholm (Dnr. 2017/799-31). All study subjects provided oral and written consent prior to participation.

### Study Subjects

Thirty MDD patients were included, all with a current episode treated with an SSRI without response for a minimum of 4 weeks. Subjects were randomly allocated in a ketamine (*n* = 20, mean age ± SD: 39.2 ± 11.55; females/males: 8/12) or a placebo group (*n* = 10, mean age ± SD: 37.1 ± 11.18; females/males: 6/4). There were no significant differences in demographics for the study subjects. Subjects in the placebo group had higher baseline Montgomery–Åsberg Depression Rating Scale (MADRS) scores than subjects in the ketamine group (30.8 vs 26.3, respectively, *p* = 0.046). The patients were diagnosed based on the International Statistical Classification of Diseases and Related Health Problems 10th Revision (ICD-10) using a Mini International Neuropsychiatric Interview (M.I.N.I.) (Sheehan et al., 1998). Main exclusion criteria included bipolar disorder, psychosis, neurodevelopmental disorders, other comorbid disorder as primary diagnoses, substance abuse, ongoing fluoxetine treatment, contraindications to ketamine treatment, and obesity or body weight ≥100 kg.

### Study Design

A wash-out period of at least five drug half-lives took place prior to the study. At baseline, venous blood samples were collected into vacutainer tubes containing EDTA and severity of depression was assessed within the same day using MADRS. Within 2 days, subjects received 40 min intravenous infusion of racemic ketamine 0.5 mg/kg diluted in 100 mL isotonic NaCl solution (ketamine group) or 100 mL isotonic NaCl solution only (placebo group). Subjects were blinded to treatment allocation. After 1–2 days, a second venous blood sample was collected and MADRS was assessed (MADRS2). All subjects were offered an open label ketamine treatment series, to be received within 2 weeks, resulting in a maximum of four ketamine treatments in total. MADRS3 was assessed directly after the last ketamine treatment. One subject chose to leave the study in the open-label phase, resulting in 19 subjects in the ketamine group who underwent all MADRS examinations.

For evaluation of changes in depressive symptoms, a shortened MADRS (MADRS-short) was used, in which the items of appetite and sleep were excluded, as preregistered at Aspredicted.org (#1760.2). This was chosen based on to the fact that ketamine can cause insomnia and lack of appetite. Response to treatment was quantified as a reduction of >50% of the MADRS score from baseline to MADRS2 or MADRS3. MADRS2 scores in the ketamine group were significantly reduced compared to MADRS1 (p_adjusted_ < 0.001). Moreover, MADRS2 scores in the ketamine group were lower compared to MADRS2 scores in the placebo group (p_adjusted_ = 0.019). Overall, a significant reduction in MADRS score was seen from baseline to MADRS3 (p_adjusted_ < 0.001).

### Plasma Extraction

Collected blood samples were processed within 2 h at 4°C to reduce variability for measurement of BDNF levels (Tsuchimine et al., 2014). Samples were centrifuged at 1500 × *g* for 15 min. Plasma supernatant was then transferred to a new sterile microfuge tube and received another centrifugation at 10,000 × *g* for 10 min, in order to completely separate platelets and prevent the release of BDNF from platelets. Plasma supernatant was then transferred to a new sterile microfuge tube and stored at −80°C until assay.

### Enzyme Linked Immunosorbent Assay

Plasma mBDNF and S100B were measured by enzyme linked immunosorbent assay kits, BDNF (Aviscera Bioscience, Cat #: SK00752-01) and S100B (Merck Millipore, Cat #: EZHS100B-33K), following the manufacturers’ instructions. All the samples were analyzed in duplicate. S100B levels in five patients (four in ketamine group and one in placebo group) were undetectable, since the values fell below the minimum detectable threshold of the kit. Therefore data on S100B levels of these subjects were excluded from the analysis.

### Statistical Analysis

Data was analyzed using GraphPad Prism version 8 (GraphPad Software, La Jolla, CA, United States^1^) and figures were created using R Studio (version 3.6.3, R Development Core Team, 2020). Distributions of all the variables were checked with the Shapiro–Wilk normality test. The Mann–Whitney test was used for between-group comparisons and Wilcoxon signed rank test for paired samples was conducted for within-group comparisons. The correlations between biological data and MADRS scores were calculated using the Spearman method. Correction for multiple comparisons was carried out using the Bonferroni method.

## Results

### Plasma BDNF and S100B Concentrations in Ketamine and Placebo Group

**FIGURE 1:**
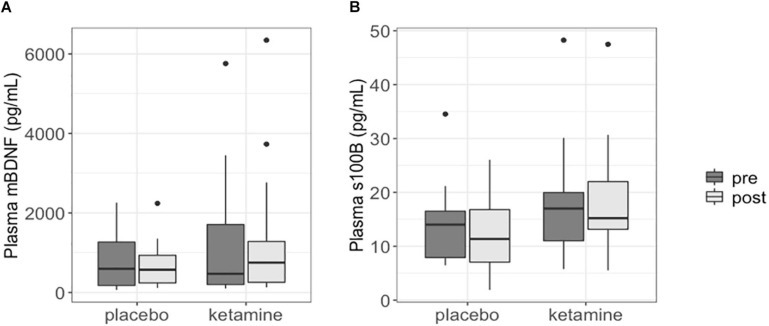
Boxplots showing plasma mBDNF **(A)** and S100B **(B)** levels in patients with SSRI-resistant major depressive disorder. Each box represents the interquartile range of the data, with median shown as horizontal line. Vertical lines represent the minimum and maximum values within 1.5 times the interquartile range distance from the 1st and 3rd quartile, respectively. Outliers are represented as single dots.

Changes in plasma mBDNF and S100B levels were not significantly different between the ketamine group and placebo group (*p* = 0.589 and *p* = 0.251, respectively). Plasma BDNF and S100B levels at baseline did not significantly differ between the two study groups (BDNF: *p* = 0.559; S100B: *p* = 0.452). Moreover, plasma mBDNF and S100B levels were not significantly different between pre-treatment and post-treatment in both the ketamine (BDNF: *p* = 0.756; S100B: *p* = 0.404) and placebo group (BDNF: *p* = 0.846; S100B: *p* = 0.652) (Figure 1).

Plasma mBDNF and S100B levels did not significantly change in responders to ketamine treatment (*n* = 15; BDNF: *p* = 0.950; S100B: *p* = 0.503), nor did post ketamine protein levels differ between responders and non-responders (*n* = 5) when corrected for baseline levels, age and gender (BDNF: *p* = 0.563; S100B: *p* = 0.832).

### Correlations Between Plasma mBDNF and S100B, and MADRS Scores

**FIGURE 2:**
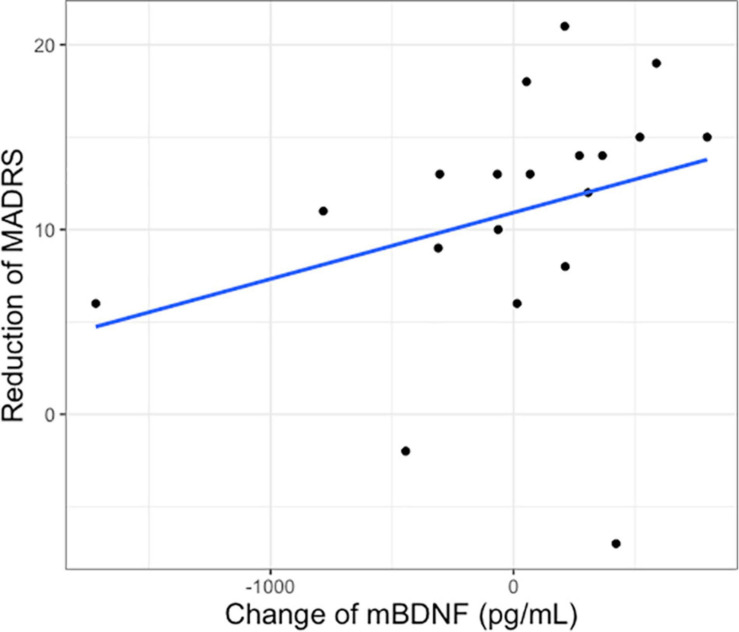
Change of plasma mBDNF after first intervention and reduction of Montgomery–Åsberg Depression Rating Scale score after 2 weeks of treatment (MADRS1 to 3) in the ketamine group.

There was no significant correlation between baseline mBDNF (rho = −0.151, *p* = 0.426) or S100B (rho = −0.121, *p* = 0.565) and baseline MADRS1. The change of plasma mBDNF after intervention in the ketamine group was positively correlated with the reduction of MADRS1 to MADRS3 (rho = 0.495, *p* = 0.031, p_adjusted_ = 0.062) (Figure 2), but not correlated with MADRS after first intervention (MADRS1 vs MADRS2) (rho = 0.301, *p* = 0.197). No significant differences were found between the change of mBDNF and MADRS changes in the placebo group (rho = −0.176, *p* = 0.626). There were also no significant correlations between changes of S100B and changes of MADRS in the ketamine group (MADRS1 vs MADRS2: rho = 0.259, *p* = 0.333; MADRS1 vs MADRS 3: rho = 0.083, *p* = 0.761).

## Discussion

In this study, we did not see a significant change of plasma mBDNF and S100B levels after 1–2 days of single ketamine compared to placebo. Moreover, plasma mBDNF and S100B levels did not significantly differ between responders and non-responders following ketamine. Change of plasma mBDNF after 1–2 days of blinded single ketamine treatment was positively correlated with reductions of MADRS scores after multiple ketamine treatments in the open-label phase. However, since this significance would not survive correction for multiple corrections this finding should be taken with caution. Changes in S100B levels did not significantly correlate with changes in MADRS scores.

Brain-derived neurotrophic factor has previously been shown to be essential for the fast acting (within hours) and sustained (up to 7 days) antidepressant response of ketamine (Autry et al., 2011; Laje et al., 2012; Liu et al., 2012). Blood BDNF concentrations are considered to reflect brain concentrations as a positive correlation of BDNF levels between brain tissue and blood was demonstrated in preclinical clinical studies (Sartorius et al., 2009; Klein et al., 2011). Our results do not indicate that ketamine administration increases plasma BDNF levels as previously reported (Duncan et al., 2013; Haile et al., 2014), but are in line with other studies which do not see a significant effect of ketamine (Machado-Vieira et al., 2009; Medeiros et al., 2021). Differences between our results and those of pre-clinical studies can be a result of the higher dose of ketamine administrated in the pre-clinical studies. The inconsistency of the previously published clinical studies and our results can be explained by several other factors. First of all, in our study plasma BDNF levels are measured in a time frame representing the sustained anti-depressant action of ketamine (1–2 days). Most studies focus on the rapid effect (within 4 h) of ketamine, while the only other study measuring plasma BDNF in this time frame also do not find a significant effect (Medeiros et al., 2021). Second, differences in analysis method might have a strong effect on outcomes for plasma BDNF levels. It has been suggested that only recent detection methods have been able to distinguish mBDNF from its precursor (Hashimoto, 2016), therefore previous results might partly reflect an effect on proBDNF. Moreover, plasma BDNF levels has been shown to be highly affected by the centrifugation strategy to extract plasma samples (Gejl et al., 2019). While in our study BDNF is measured in platelet poor plasma, other studies where a second centrifugation step was not applied might have been affected by BDNF-release by platelets (Gejl et al., 2019). Third, the design of the studies is different in the previously mentioned studies. Our results are in line with a placebo-controlled double blind study (Medeiros et al., 2021). Finally, the MDD patient population in our study were specifically SSRI-resistant, while the other studies investigated the effect of ketamine in TRD, defined as non-response to 1–3 antidepressant treatments. Our results suggest that the previously reported lack of association applies not only to MDD patients who are considered treatment-resistant, but also specifically to MDD patients who are treatment-resistant to SSRIs. Further studies should investigate if this would apply to the whole MDD population when treated with ketamine.

Our results show that a change of plasma BDNF after 1–2 days of ketamine was positively correlated with clinical improvement on MADRS as assessed after 1–2 weeks of series ketamine infusion. This is consistent with previous studies reporting a correlation between plasma BDNF levels and rapid response of ketamine antidepressant, suggesting that BDNF is a potential response biomarker (Duman et al., 2012; Haile et al., 2014) and suggests that early change of plasma mBDNF can predict antidepressant response in patients with SSRI-resistant depression. However, as the significant correlation does not survive correction for multiple comparisons, this result should be taken with caution.

Our data showed no significant change of S100B levels induced by ketamine, despite the observed clinical improvement on MADRS scores. Moreover, we did not see a significant correlation between S100B levels or changes and treatment response. Previous studies suggest a role of peripheral S100B in response to various other antidepressant treatments (Arolt et al., 2003; Schroeter et al., 2013; Ambrée et al., 2015). Our results suggest that this association might be lacking for the antidepressant response to ketamine treatment and that change of plasma S100B levels might not be critical for clinical improvement in MDD patients. According to our data, there was no significant correlation between S100B levels and MADRS levels before intervention, suggesting that plasma S100B may not be a state marker for depression severity. Although elevated levels of S100B in MDD patients (Schroeter et al., 2013; Shi et al., 2020) and positive correlations between S100B levels and depression severity (Schroeter et al., 2013) have been reported, also reduced levels have been found in cerebrospinal fluid of depressed subjects (Uher and Bob, 2012). Several reasons may account for this inconsistency. First, peripheral S100B may also be affected by extracranial sources, as S100B is also expressed in the peripheral tissues including white fat, skeletal muscle, or heart (Zimmer et al., 1995). This is especially of importance in patients with comorbid obesity, heart disease or metabolic syndrome (Mazzini et al., 2005, 2007, 2009; Steiner et al., 2010a,b). One study showed that subjects with a BMI >30 had markedly higher S100B levels when compared with a lower BMI (Steiner et al., 2010a). In our study, BMI levels were similar in both intervention groups. Second, as an immune-modulator, S100B concentrations may be affected by immune function, which is known to be dysregulated in depression. To reach the goal of sufficient prediction of treatment response, detecting multiple anti- and pro-inflammatory factors to form a diagnostic platform is necessary in the future.

There are some limitations to the present study. First, the number of subjects in our study was limited to that of the primary PET study, which resulted in a relatively small sample size. This might specifically have affected the analysis on S100B levels as measurements of samples from five subjects provided values below a reliably detectable level. To limit the effect on power, the data was also analyzed using a linear mixed model with time and treatment group as fixed effects and subject as random effect. However, this did not alter our conclusions. Secondly, the time frame of measurement after intervention might increase heterogeneity of the samples while the number of measurement occasions was limited to two time points.

## Conclusion

Taken together, our findings add up to results from previous studies which did not show a critical role for increased plasma mBDNF for the sustained antidepressant effect of ketamine in MDD patients. Early changes of plasma mBDNF levels might predict the likelihood of the sustained antidepressant response to ketamine treatment, however, this should be further studied. No significant effect of ketamine administration on S100B levels and no association with treatment response was seen.

## Data Availability Statement

The individual data underlying the results presented in this article are not readily available due to EU legislation and the available ethical permit for this study. This study was part of a larger study, in which participants did not provide informed consent on data sharing. Requests for information on other aspects of the study, such as analysis protocols, can be directed to the corresponding author.

## Ethics Statement

The studies involving human participants were reviewed and approved by the Regional Ethical Review Board in Stockholm. The patients/participants provided their written informed consent to participate in this study.

## Author Contributions

HJ and EV planned and performed the experimental work and analyses. HJ wrote the first draft of the manuscript and EV performed the secondary writing and editing. PS and JL managed study design. MT and C-JE were responsible for recruitment and examination of the patients. All authors contributed to and have approved the final manuscript.

## Conflict of Interest

The authors declare that the research was conducted in the absence of any commercial or financial relationships that could be construed as a potential conflict of interest.

## Footnotes

1 www.graphpad.com
